# Nobiletin Decreases Inflammatory Mediator Expression in Tumor Necrosis Factor-Stimulated Human Periodontal Ligament Cells

**DOI:** 10.1155/2021/5535844

**Published:** 2021-07-10

**Authors:** Yoshitaka Hosokawa, Ikuko Hosokawa, Kazumi Ozaki

**Affiliations:** ^1^Department of Conservative Dentistry, Institute of Biomedical Sciences, Tokushima University Graduate School, Tokushima, Tokushima, Japan; ^2^Department of Oral Health Care Promotion, Institute of Biomedical Sciences, Tokushima University Graduate School, Tokushima, Tokushima, Japan

## Abstract

Nobiletin, a biologically active substance in the skin of citrus fruits, has been reported to be an effective anti-inflammatory, anticancer, and antimicrobial agent. In this study, we aimed to examine the anti-inflammatory effects of nobiletin on tumor necrosis factor- (TNF-) stimulated human periodontal ligament cells (HPDLCs). Our results demonstrated that nobiletin treatment could decrease the expressions of inflammatory cytokines (C-X-C motif chemokine ligand (CXCL)10, C-C motif chemokine ligand (CCL)2, and interleukin- (IL-) 8), matrix metalloproteinases (MMPs) (MMP1 and MMP3), and prostaglandin-endoperoxide synthase 2 (PTGS2) in TNF-stimulated HPDLCs. Moreover, we revealed that nobiletin could inhibit the activation of nuclear factor- (NF-) *κ*B and protein kinase B (AKT1) pathways in TNF-stimulated HPDLCs. Furthermore, nobiletin treatment enhanced nuclear factor, erythroid 2 like 2 (NFE2L2) and heme oxygenase 1 (HMOX1) expressions in TNF-stimulated HPDLCs. In conclusion, these findings suggest that nobiletin can inhibit inflammatory responses in TNF-stimulated HPDLCs by inhibiting NF-*κ*B and AKT1 activations and upregulating the NFE2L2 and HMOX1 expression.

## 1. Introduction

Pathogenic bacteria of periodontal disease could induce alveolar bone resorption in periodontal lesions. The excessive immune reaction is related to periodontal tissue destruction. Tumor necrosis factor (TNF) (also known as TNF-*α*) is positively involved in the pathogenesis of periodontal disease because it could induce chemokines, MMPs, and prostaglandin E2 (PGE2) production in periodontal resident cells including human periodontal ligament cells (HPDLCs) [1–3]. We previously reported that TNF could inhibit some kinds of chemokines and matrix metalloproteinase (MMP)s in HPDLCs [4–6]. Ransjö et al. also found that TNF treatment enhanced PGE2 production in HPDLCs [7]. So, it is important to find the bioactive substance that could inhibit the influence of TNF on HPDLCs because TNF is the main inducer of inflammation in periodontal lesions.

**Figure 1:**
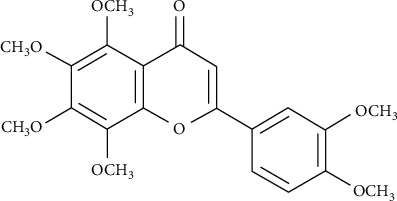
The chemical structure of nobiletin.

Nobiletin is a biologically active substance in the skin of citrus fruits, such as *C. sinensis* (sweet orange), *C. aurantium L*. (sour orange), and *C. paradisi* (grapefruit). Also, citrus fruit juice contains detectable amounts (1–10 mg/g) of nobiletin [8]. The structure of nobiletin is shown in Figure 1. Previous studies have demonstrated a wide range of beneficial activities of nobiletin, including anti-inflammation [9], anticancer [10], and antibacterial activities [11]. For example, it is reported that nobiletin inhibited interferon alpha 1 and interferon beta 1 release in lipopolysaccharide or CpG oligodeoxynucleotides treated prostate cancer cells [9]. Nobiletin inhibited several of the hallmark features of colorectal cancer pathophysiology, including arresting the cell cycle and inhibiting cell proliferation [10]. However, few researchers are going to use nobiletin for the treatment of periodontal disease.

We decided to perform this research as a basic study to use nobiletin for the prevention or treatment of periodontitis. In other words, we examined the effects of nobiletin on the expressions of inflammatory cytokines, MMPs, and prostaglandin-endoperoxide synthase 2 (PTGS2) in TNF-stimulated HPDLCs which are the main constituents of the periodontium. In addition, we investigated to confirm the influence of nobiletin to give the activation of the signal transduction pathways that TNF stimulation derived.

## 2. Materials and Methods

### 2.1. Reagents

Nobiletin (item number: 15421) and the antibody against PTGS2 (item number: 10112) were purchased from Cayman Chemical (Ann Arbor, MI, USA). Recombinant human TNF(catalog number: 300-01A) was obtained from PeproTech (Rocky Hill, NJ, USA). Antibodies against phosphomitogen activated protein kinase (MAPK)14 (#4551), phospho-MAPK1/MAPK3 (#4370), phospho-MAPK8 (#4668), phospho-IkappaB kinase- (IKK-) *α*/*β* (#2697), phosphonuclear factor- (NF-) *κ*B p65 (#3033), phosphoprotein kinase B (AKT1) (#4060), MAPK14 (#9212), MAPK1/MAPK3 (#4695), MAPK8 (#9252), IKK-*α* (#2682), NF-*κ*B p65 (#8242), AKT1 (#2920), heme oxygenase 1 (HMOX1) (#5853), nuclear factor, erythroid 2 like 2 (NFE2L2) (#12721), or glyceraldehyde-3-phosphate dehydrogenase (GAPDH) (#5174) were purchased from Cell Signaling Technology (Danvers, MA, USA). Enzyme-linked immunosorbent assay (ELISA) kits of CXCL10 (catalog number: DY266), CCL2 (catalog number: DY279), IL-8 (catalog number: DY208), MMP1 (catalog number: DY901), MMP3 (catalog number: DY513), and TIMP1 (catalog number: DY970) were purchased from R&D Systems (Minneapolis, MN, USA).

### 2.2. Cell Culture

HPDLCs were purchased from Lonza Japan (Tokyo, Japan) and cultured in Dulbecco's minimal essential medium (DMEM, Gibco, Grand Island, NY, USA) containing 10% fetal bovine serum (FBS, Gibco), 100 U/ml penicillin, and 100 *μ*g/ml streptomycin (Invitrogen) in a 5% CO_2_ incubator at 37°C. HPDLCs were seeded in 24-well plates or 12-well plates at 5000 cells per cm^2^.

### 2.3. Determination of Cytokines and MMP Secretion with ELISA

The release of CXCL10, CCL2, IL-8, MMP1, MMP3, and TIMP1 in the culture medium was tested by ELISA kits. HPDLCs were seeded in a 24-well culture plate. HPDLCs were incubated with different concentrations of nobiletin (12.5, 25, 50, or 100 *μ*M) and then stimulated with TNF (10 ng/ml) for 24 hours. Then, cell supernatants were collected and used for ELISA analysis. All assays were performed according to the manufacturer's protocols, and CXCL10, CCL2, IL-8, MMP1, MMP3, and TIMP1 levels were determined using the standard curve prepared for each assay. The experiments were performed in triplicate and repeated three times.

### 2.4. Western Blot Analysis

HPDLCs were pretreated with nobiletin (25, 50, or 100 *μ*M) for 1 hour before the stimulation with TNF (10 ng/ml) for 15, 30, or 60 min (analysis for the activation of signal transduction pathway) or 24 hours (analysis for the PTGS2, HMOX1, and NFE2L2 expression). Then, the cell supernatant was discarded and washed twice with cold phosphate-buffered saline (PBS). HPDLCs were lysed with cell lysis buffer (Cell Signaling Technology) including protease inhibitor cocktail (Sigma, St. Louis, MO, USA). Debris was removed by centrifugation at 15000 rpm for 10 min at 4°C, and the supernatants were collected. Equal amounts of protein were separated by sodium dodecyl sulfate-polyacrylamide gel electrophoresis (SDS-PAGE) and transferred to polyvinylidene difluoride (PVDF) membranes (Millipore, Bradford, USA). The membranes were blocked with 1% skim milk for 1 hour at room temperature. Then, the membranes were incubated with the following primary antibodies at 4°C overnight: phospho-MAPK14 (1 : 1000), phospho-MAPK1/MAPK3 (1 : 2000), phospho-MAPK8 (1 : 1000), phospho-IKK-*α*/*β*(1 : 1000), phospho-NF-*κ*B p65 (1 : 1000), phosph-AKT1 (1 : 2000), MAPK14 (1 : 1000), MAPK1/MAPK3 (1 : 2000), MAPK8 (1 : 1000), IKK-*α*(1 : 1000), NF-*κ*B p65 (1 : 1000), AKT1 (1 : 2000), PTGS2 (1 : 1000), HMOX1 (1 : 1000), NFE2L2 (1 : 1000), or GAPDH (1 : 8000). The membranes were washed with Tris-buffered saline including 0.1% Tween 20 (TBS-T) 3 times and exposed to horseradish peroxidase-conjugated secondary antibodies (Sigma) for 1 hour at room temperature. The membranes were washed with TBS-T 3 times, and the protein bands were visualized by the Enhanced Chemiluminescence (ECL) Plus Western blotting detection system (GE Healthcare, Uppsala, Sweden) according to the manufacturer's instructions. The experiments were repeated three times. The densities of bands of Western blot analysis were determined using ImageJ software (NIH, Bethesda, MD, USA).

### 2.5. Statistical Analysis

Statistical differences between the means of the sample groups were calculated by the Kolmogorov-Smirnov test and the one-way analysis of variance (ANOVA) followed by Tukey's test. Any statistically significant difference between the groups was determined at the *p* < 0.05 level.

## 3. Results

### 3.1. Nobiletin Inhibits Chemokine Production in TNF-Stimulated HPDLCs

**Figure 2:**
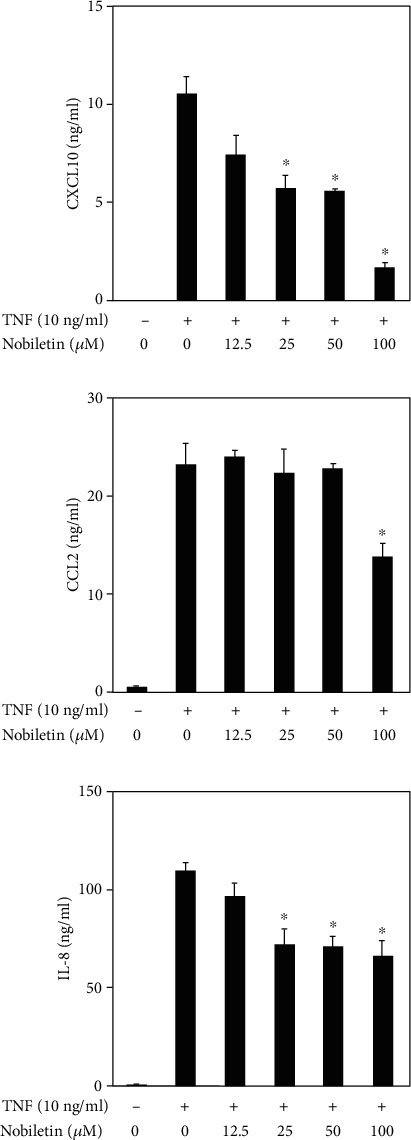
Effects of nobiletin on TNF-induced CXCL10, CCL2, and IL-8 production in HPDLCs. HPDLCs were cultured with TNF (10 ng/ml) and nobiletin (12.5, 25, 50, or 100 *μ*M) for 24 hours. The amounts of CXCL10, CCL2, and IL-8 in the supernatants were determined using their respective ELISA kits. Bars represent the mean ± standard deviation (SD) of data from three independent experiments. ^∗^*p* < 0.01: significantly different from the result for the TNF-stimulated HPDLCs that were not treated with nobiletin.

To investigate the anti-inflammatory effects of nobiletin on TNF-stimulated HPDLCs, production of CXCL10, CCL2, and IL-8 was detected by ELISA assay (Figure 2). Levels of these chemokines were significantly upregulated by TNF stimulation, compared with the control. However, nobiletin suppressed the TNF-induced production of CXCL10 and IL-8 in a dose-dependent manner (Figure 2). A high concentration of nobiletin (100 *μ*M) significantly decreased CCL2 production in TNF-stimulated HPDLCs. We tested the cytotoxic effects of nobiletin (100 *μ*M) on HPDLCs using Cell Counting Regents (Nacalai Tesque, Kyoto, Japan).

### 3.2. Nobiletin Inhibited MMP1 and MMP3 Production in TNF-Stimulated HPDLCs

**Figure 3:**
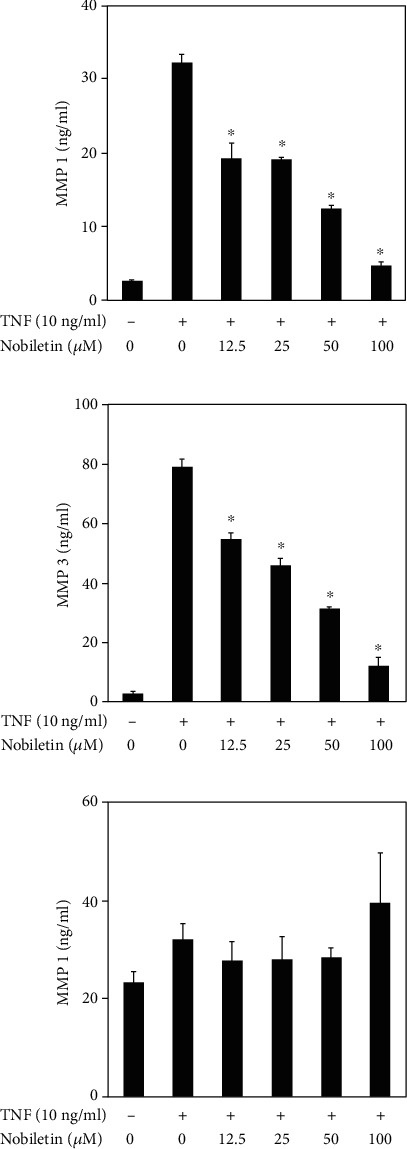
Effects of nobiletin on TNF-induced MMP1, MMP3, and TIMP1 production in HPDLCs. HPDLCs were cultured with TNF (10 ng/ml) and nobiletin (12.5, 25, 50, or 100 *μ*M) for 24 hours. The amounts of MMP1, MMP3, and TIMP1 in the supernatants were determined using their respective ELISA kits. Bars represent the mean ± SD of data from three independent experiments. ^∗^*p* < 0.01: significantly different from the result for the TNF-stimulated HPDLCs that were treated without nobiletin.

The release of MMP1 and MMP3 in culture supernatants induced by TNF in the presence of nobiletin was determined by ELISA. As shown in Figure 3, nobiletin treatment significantly inhibited TNF-induced MMP1 and MMP3 production in HPDLCs in a dose-dependent manner. On the other hand, nobiletin treatment did not change TIMP1 production in TNF-stimulated HPDLCs.

### 3.3. The Effect of Nobiletin on PTGS2 Expression in TNF-Stimulated HPDLCs

**Figure 4:**
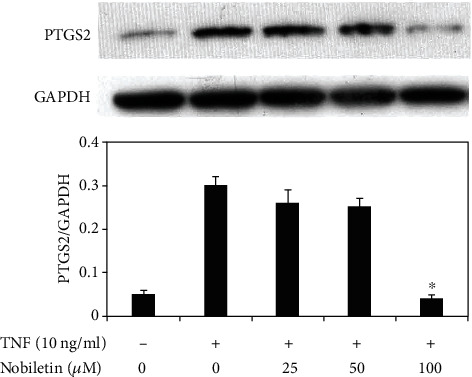
Effects of nobiletin on TNF-induced PTGS2 expression in HPDLCs. HPDLCs were stimulated by TNF (10 ng/ml) with or without nobiletin (12.5, 25, or 50 *μ*M) for 24 hours, and then PTGS2 expression was determined by Western blot analysis. Each photograph is representative of the results of 3 separate experiments. The quantification was performed using image analysis software. ^∗^*p* < 0.05 compared to the TNF stimulation without nobiletin.

To assess the influence of nobiletin on the expression of PTGS2, Western blot analysis was performed (Figure 4). The treatment with TNF significantly increased the expression of PTGS2 compared to untreated controls. Figure 4 also shows that PTGS2 expression enhanced by TNF stimulation was decreased by 100 *μ*M nobiletin treatment.

### 3.4. Effects of Nobiletin on MAPKs, NF-*κ*B, and AKT1 Activation in TNF-Stimulated HPDLCs

**Figure 5:**
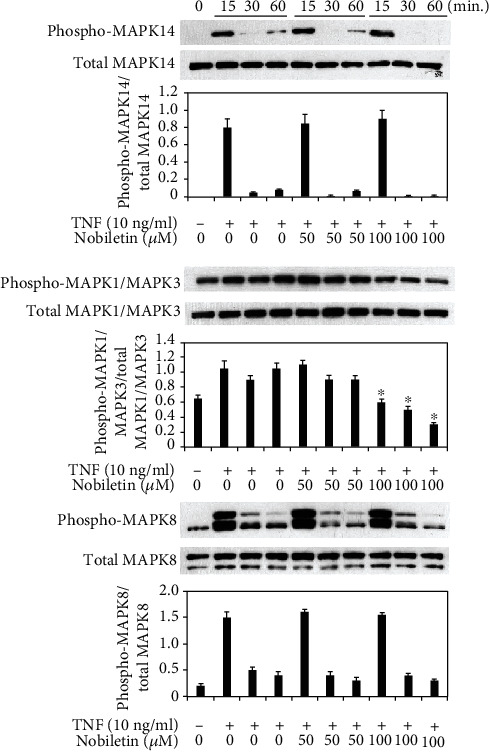
Nobiletin mediated inhibition of MAPK pathways in TNF-stimulated HPDLCs. After pretreatment with nobiletin (50 or 100 *μ*M) for 1 hour, HPDLCs were stimulated with TNF (10 ng/ml) for 15, 30, or 60 minutes, and then phosphorylation of MAPK14, MAPK1/MAPK3, and MAPK8 was determined by Western blot analysis. Each photograph is representative of the results of 3 separate experiments. The quantification was performed using image analysis software. ^∗^*p* < 0.05 compared to the TNF stimulation without nobiletin.

**Figure 6:**
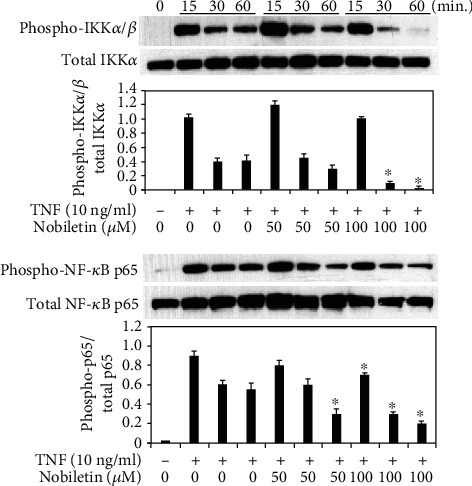
Nobiletin mediated inhibition of the NF-*κ*B pathway in TNF-stimulated HPDLCs. After pretreatment with nobiletin (50 or 100 *μ*M) for 1 hour, HPDLCs were stimulated with TNF (10 ng/ml) for 15, 30, or 60 minutes, and then phosphorylation of IKK-*α*/*β* and NF-*κ*B p65 was determined by Western blot analysis. Each photograph is representative of the results of 3 separate experiments. The quantification was performed using image analysis software. ^∗^*p* < 0.05 compared to the TNF stimulation without nobiletin.

**Figure 7:**
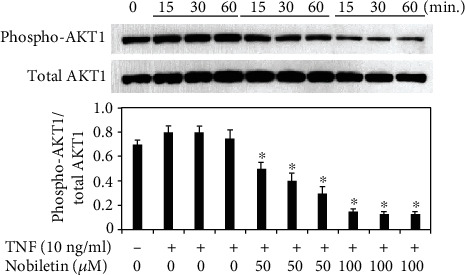
Nobiletin mediated inhibition of AKT1 in TNF-stimulated HPDLCs. After pretreatment with nobiletin (50 or 100 *μ*M) for 1 hour, HPDLCs were stimulated with TNF (10 ng/ml) for 15, 30, or 60 minutes, and then phosphorylation of AKT1 was determined by Western blot analysis. Each photograph is representative of the results of 3 separate experiments. The quantification was performed using image analysis software. ^∗^*p* < 0.05 compared to the TNF stimulation without nobiletin.

Next, we examined if nobiletin modified activations of signal transduction pathways in TNF-stimulated HPDLCs. We previously reported that TNF treatment could activate MAPK14, MAPK1/MAPK3, MAPK8, NF-*κ*B, and AKT1 in HPDLCs [4, 5, 12]. Therefore, we used Western blot analysis to detect the effects of nobiletin on activations of the signal transduction pathway. Figures 5 and 6 show that 100 *μ*M nobiletin treatment inhibited the phosphorylation levels of MAPK1/MAPK3, IKK-*α*/*β*, and p65 NF-*κ*B in TNF-stimulated HPDLCs. The level of AKT1 phosphorylation was slightly decreased by 50 *μ*M nobiletin treatment, and 100 *μ*M nobiletin inhibited AKT1 phosphorylation in TNF-treated HPDLCs (Figure 7).

### 3.5. The Effect of Nobiletin on HMOX1 and NFE2L2 Expression in TNF-Stimulated HPDLCs

**Figure 8:**
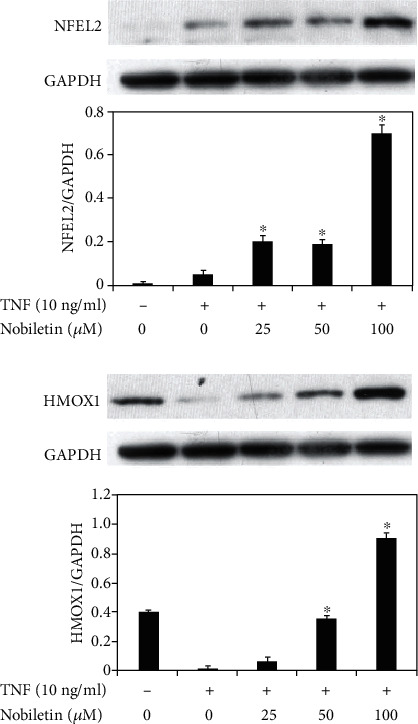
Effects of nobiletin on NFE2L2 and HMOX1 expression in TNF-stimulated HPDLCs. HPDLCs were stimulated by TNF (10 ng/ml) with or without nobiletin (25, 50, or 100 *μ*M) for 24 hours, and then NFE2L2 and HMOX1 expression was determined by Western blot analysis. Each photograph is representative of the results of 3 separate experiments. The quantification was performed using image analysis software. ^∗^*p* < 0.05 compared to the TNF stimulation without nobiletin.

It is known that HMOX1 has anti-inflammatory roles in inflammatory lesions [13], and NFE2L2 could induce HMOX1 expression [14]. So, we focus on the effects of nobiletin on HMOX1 and NFE2L2 expression in HPDLCs. As shown in Figure 8, TNF treatment inhibited HMOX1 expression in HPDLCs. On the other hand, nobiletin treatment rescued HMOX1 expression in TNF-stimulated HPDLCs in a dose-dependent manner. Figure 8 also shows that nobiletin could enhance NFE2L2 expression in HPDLCs in a concentration-dependent fashion.

## 4. Discussion

Periodontal disease is the most popular infectious disease in the world that cuts down on the quality of life by losing teeth. The strong immune reaction in periodontal lesions is related to periodontium destruction. Therefore, the discovery of a safe bioactive substance which we can give in the periodontal lesion is hoped for. It is already reported that nobiletin has anti-inflammatory effects. For example, Liu et al. reported that nobiletin could inhibit IL-6, TNF, MMP1, and MMP3 in interleukin-21-stimulated human synoviocytes [15]. Lin et al. also reported that nobiletin could decrease PGE2 and PTGS2 expression in IL-1B-treated human synovial fibroblasts [16]. In this report, we showed that nobiletin could inhibit inflammatory chemokines, MMPs, and PTGS2 expression in TNF-stimulated HPDLCs. Previous reports and this report explain that nobiletin has anti-inflammatory effects on various types of cells.

MAPK, NF-*κ*B, and AKT1 pathways are involved in inflammatory mediator production. Therefore, previous researchers examined the effects of nobiletin on activations of signal transduction pathways. Shi et al. reported that nobiletin could inhibit MAPK1/MAPK3 and AKT1 phosphorylation in hepatocyte growth factor-treated HepG2 cells [17]. Xie et al. reported that nobiletin dramatically suppressed the IL-1B-stimulated phosphorylation of AKT1 and activation of NF-*κ*B in human chondrocytes [18]. Results of previous reports are similar to this report. The inhibitory effects of nobiletin on MAPK1/MAPK3, AKT1, and NF-*κ*B activations might be involved in the inhibitory effects of inflammatory mediator expressions in various types of cells.

To better understand the underlying molecular mechanisms, the NFE2L2-HMOX1 pathway was investigated. HMOX1 could inhibit NF-*κ*B activation, and NFE2L2 is related to HMOX1 production [19]. Therefore, we focus on NFE2L2 and HMOX1 expression in nobiletin-treated HPDLCs because we got the information that nobiletin could inhibit NF-*κ*B activation in this study (Figure 6). In this report, we revealed that nobiletin treatment enhanced HMOX1 and NFE2L2 expression in human periodontal ligament cells. Based on these findings, we hypothesize that enhancement of HMOX1 expression in nobiletin-treated HPDLCs might decrease the activation level of NF-*κ*B in TNF-stimulated HPDLCs.

In conclusion, this study demonstrates the anti-inflammatory activity of nobiletin in HPDLCs. Nobiletin significantly inhibits the TNF-induced inflammatory response by suppressing the MAPK1/MAPK3, NF-*κ*B, and AKT1 signaling pathways and enhancing HMOX1 and NFE2L2 expression. We should use nobiletin for animal models of periodontal disease at the next stage. If nobiletin could prevent the progression of periodontal disease in animal diseased models, we may use nobiletin for human periodontal disease treatment. We think nobiletin might be used for local drug delivery systems such as periodontal pocket irrigation or gel application in periodontal lesions. Further studies should be necessary to prove the hypothesis.

## Data Availability

The data used to support the findings of this study are available from the corresponding author upon request.

## References

[1] Graves D. T. (1999). The potential role of chemokines and inflammatory cytokines in periodontal disease progression. *Clinical Infectious Diseases*.

[2] Birkedal-Hansen H. (1993). Role of matrix metalloproteinases in human periodontal diseases. *Journal of Periodontology*.

[3] Page R. C. (1991). The role of inflammatory mediators in the pathogenesis of periodontal disease. *Journal of Periodontal Research*.

[4] Hosokawa I., Hosokawa Y., Ozaki K., Matsuo T. (2020). Carnosic acid inhibits inflammatory cytokines production in human periodontal ligament cells. *Immunopharmacology and Immunotoxicology*.

[5] Hosokawa Y., Hosokawa I., Ozaki K., Matsuo T. (2019). Sudachitin inhibits matrix metalloproteinase-1 and -3 production in tumor necrosis factor-*α*-stimulated human periodontal ligament cells. *Inflammation*.

[6] Shindo S., Hosokawa Y., Hosokawa I., Ozaki K., Matsuo T. (2014). Genipin inhibits MMP-1 and MMP-3 release from TNF-*α*-stimulated human periodontal ligament cells. *Biochimie*.

[7] Ransjö M., Marklund M., Persson M., Lerner U. H. (1998). Synergistic interactions of bradykinin, thrombin, interleukin 1 and tumor necrosis factor on prostanoid biosynthesis in human periodontal-ligament cells. *Archives of Oral Biology*.

[8] Dugo P., Mondello L., Dugo L., Stancanelli R., Dugo G. (2000). LC-MS for the identification of oxygen heterocyclic compounds in citrus essential oils. *Journal of Pharmaceutical and Biomedical Analysis*.

[9] Deveci Ozkan A., Kaleli S., Onen H. I. (2020). Anti-inflammatory effects of nobiletin on TLR4/TRIF/IRF3 and TLR9/IRF7 signaling pathways in prostate cancer cells. *Immunopharmacology and Immunotoxicology*.

[10] Goh J. X. H., Tan L. T., Goh J. K. (2019). Nobiletin and derivatives: functional compounds from citrus fruit peel for colon cancer chemoprevention. *Cancers (Basel)*.

[11] Wu T., Zang X., He M., Pan S., Xu X. (2013). Structure-activity relationship of flavonoids on their anti-Escherichia coli activity and inhibition of DNA gyrase. *Journal of Agricultural and Food Chemistry*.

[12] Hosokawa Y., Hosokawa I., Shindo S., Ozaki K., Matsuo T. (2017). Gomisin N decreases inflammatory cytokine production in human periodontal ligament cells. *Inflammation*.

[13] Fernández-Fierro A., Funes S. C., Rios M., Covián C., González J., Kalergis A. M. (2021). Immune modulation by inhibitors of the HO system. *International Journal of Molecular Sciences*.

[14] Zhang X., Ding M., Zhu P. (2019). New insights into the Nrf-2/HO-1 signaling axis and its application in pediatric respiratory diseases. *Oxidative Medicine and Cellular Longevity*.

[15] Liu Z., Guo S., Dong Q. (2020). Nobiletin suppresses IL-21/IL-21 receptor-mediated inflammatory response in MH7A fibroblast-like synoviocytes (FLS): an implication in rheumatoid arthritis. *European Journal of Pharmacology*.

[16] Lin N., Sato T., Takayama Y. (2003). Novel anti-inflammatory actions of nobiletin, a citrus polymethoxy flavonoid, on human synovial fibroblasts and mouse macrophages. *Biochemical Pharmacology*.

[17] Shi M. D., Liao Y. C., Shih Y. W., Tsai L. Y. (2013). Nobiletin attenuates metastasis via both ERK and PI3K/Akt pathways in HGF- treated liver cancer HepG2 cells. *Phytomedicine*.

[18] Xie L., Xie H., Chen C., Tao Z., Zhang C., Cai L. (2019). Inhibiting the PI3K/AKT/NF-*κ*B signal pathway with nobiletin for attenuating the development of osteoarthritis: in vitro and in vivo studies. *Food & Function*.

[19] Wardyn J. D., Ponsford A. H., Sanderson C. M. (2015). Dissecting molecular cross-talk between Nrf2 and NF-*κ*B response pathways. *Biochemical Society Transactions*.

